# Successful Treatment of a Recalcitrant *Staphylococcus epidermidis* Prosthetic Knee Infection with Intraoperative Bacteriophage Therapy

**DOI:** 10.3390/ph14030231

**Published:** 2021-03-08

**Authors:** James B. Doub, Vincent Y. Ng, Eleanor Wilson, Lorenzo Corsini, Benjamin K. Chan

**Affiliations:** 1 Division of Infectious Diseases, University of Maryland School of Medicine, Baltimore, MD 21201, USA; Eleanor.Wilson@ihv.umaryland.edu; 2 Department of Orthopedic Surgery, University of Maryland School of Medicine, Baltimore, MD 21201, USA; vng@som.umaryland.edu; 3 PhagoMed Biopharma GmbH, 1110 Vienna, Austria; lorenzo.corsini@phagomed.com; 4 Department of Ecology and Evolutionary Biology, Yale University, New Haven, CT 06520, USA; b.chan@yale.edu

**Keywords:** *Staphylococcus epidermidis*, periprosthetic joint infection, bacteriophage therapy, biofilm, aplastic anemia

## Abstract

Here, we present a case of a 79-year-old female with a recalcitrant *Staphylococcal epidermidis* prosthetic knee infection that was successfully treated with a single dose of adjuvant intra-articular bacteriophage therapy after debridement and implant retention surgery. The bacteriophage used in this case, PM448, is the first ɛ^2^ bacteriophage to be used in vivo. Currently the patient is without evidence of clinical recurrence and, interestingly, the patient had also suffered from debilitating aplastic anemia for over 2 years, which is recovering since receiving adjuvant bacteriophage therapy.

## 1. Introduction

Total knee arthroplasties have improved the quality of life for countless individuals. However, when arthroplasties become infected, significant morbidity and mortality occurs [1]. The conventional treatment for chronic prosthetic joint infections (PJIs) is a 2-stage revision surgery in which the infected prosthesis is removed, and an interim antibiotic loaded spacer is placed to keep the joint stable until the infection is cleared, whereby a new prosthesis can then be implanted [1,2]. This two-step revision procedure is morbid and physically demanding for patients but has an approximate 85% success rate [2]. That said, success rates have not improved over the past decade, despite aggressive investigation of novel adjuvant therapeutics [2]. Dense bacterial biofilms, small colony variants and persister cells, which conventional antibiotics have limited ability to eradicate, make these infections arduous to treat [3]. When patients have recurrence of their infections after failing a 2-stage revision surgery, they are considered to have recalcitrant infections and repeat 2-stage revision surgeries have success rates that range widely from less than 50% to 75% [4,5]. Therefore, new novel therapeutics are drastically needed to treat PJIs.

Bacteriophage therapy has been used as an adjuvant therapeutic with conventional surgical and antibiotic therapies in several compassionate use cases to cure recalcitrant PJIs and orthopedic hardware infections [3,6,7,8]. The antibiofilm activity of bacteriophages and ability to kill metabolically inactive biofilm bacteria make this a potentially attractive adjuvant treatment in PJIs [9,10]. Herein we present a unique case of a patient with a recalcitrant *Staphylococcus epidermidis* PJI that was successfully treated with adjuvant bacteriophage therapy. Interestingly, her comorbid severe aplastic anemia has also improved since receiving this therapy.

## 2. Case

79-year-old female presented to University of Maryland surgical infectious disease clinic for evaluation of her recalcitrant *S. epidermidis* PJI. Historically, the patient underwent total knee arthroplasty in 2012, for severe osteoarthritis of the left knee. This was complicated by a *S. epidermidis* PJI that was initially treated with debridement, antibiotics, irrigation and retention of the prosthesis (DAIR) surgery in which she received intravenous (IV) vancomycin for 6 weeks. However, within 6 weeks of stopping her antibiotics she had recurrence of her *S. epidermidis* PJI. This was then subsequently treated with 2-stage revision surgery and 6 weeks of IV daptomycin therapy. Reimplantation of a new knee arthroplasty was conducted but 4 weeks later she started to have erythema, worsening pain and swelling of the knee. Repeat arthrocentesis was conducted in which *S. epidermidis* was again cultured from the synovial fluid. She was then started on chronic doxycycline therapy for 6 months which did not improve her knee pain, swelling or pancytopenia.

**Figure 1:**
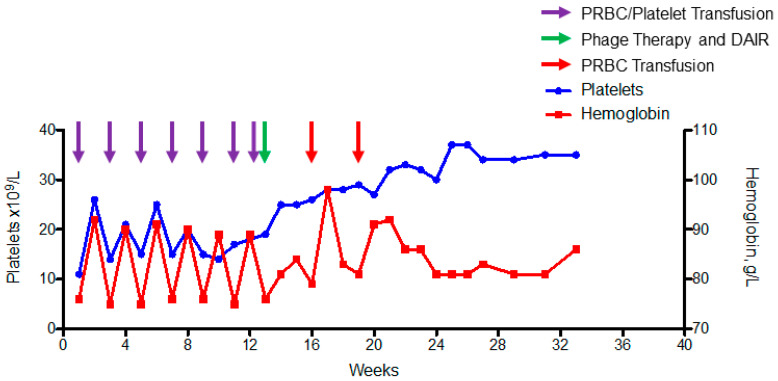
Graph of baseline hemoglobin and platelet levels showing the need for biweekly transfusions of both platelets and packed red blood cells (purple arrows) before DAIR and adjuvant bacteriophage therapy (green arrow). After bacteriophage therapy, the patient had transfusion of packed red blood cell transfusion at week 16 and 19 but no other transfusions have been required since DAIR and adjuvant bacteriophage therapy supporting bone marrow partial recovery and stabilization of levels.

Complicating this case was the patient’s severe life-threatening pancytopenia, a result of her underlying aplastic anemia from an unknown etiology that had been transfusion dependent with respect to platelets and packed red blood cells on biweekly basis for 2 years (Figure 1). She had been treated with decitabine and various doses of eltrombopag with no change in her pancytopenia. Therefore, aggressive chemotherapy (anti-thymocyte globulin and cyclosporine) was recommended to attempt to improve her aplastic anemia, but eradication of her recalcitrant PJI was deemed necessary before chemotherapy could be safely given. Repeat 2-stage arthroplasty also was deemed too high risk given her life-threatening pancytopenia. In addition, the patient declined further revision surgeries given lack of prior success. She also had no improvements while on chronic oral doxycycline therapy and, consequently, alternative adjuvant therapies were discussed.

After prolonged discussion with the patient, she elected to attempt salvage therapy with adjuvant bacteriophage therapy to be used with DAIR and IV antibiotics. A repeat arthrocentesis was obtained which again grew only *S. epidermidis*. This isolate was sent to PhagoMed in Austria to select for a highly virulent bacteriophage. Bacteriophage PM448 was identified to have lytic activity and inhibit bacterial growth of the clinical isolate. This bacteriophage was then sent to Dr. Benjamin Chan’s laboratory to make therapeutic doses. Please see methods section for more information on isolation and preparation of bacteriophage therapy.

**Figure 2:**
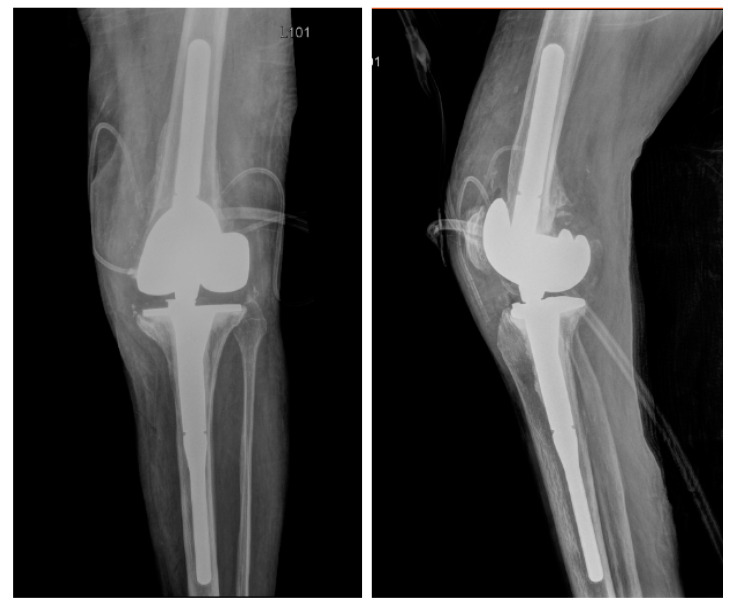
Post-operative anterior–posterior and lateral X-ray showing well fixed long stem femoral and tibial arthroplasty components. No hardware complication is present but generalized osteopenia and expected postoperative changes with intra-articular emphysema. Surgical drain is also noted.

Expanded access was granted by the FDA (IND-26759) and University of Maryland Institutional Review Board (HP-00093089). Consent for expanded access use authorization was obtained and treatment protocol was reviewed by the University of Maryland, Baltimore Institutional Review Board, who deemed this use was acceptable in accordance with 21 CFR 56.102(d) and 21 CFR 56.104(c). The patient underwent DAIR with modular exchange of the polyethylene lining by an experienced orthopedic tumor surgeon (VYN). At the end of the surgical procedure 2 × 10^10^ plaque forming units (PFU) of bacteriophage PM448 diluted in 10 mL of normal saline was injected into the intraarticular space around the prosthesis as discussed in the methods section. The patient tolerated the surgery with no significant adverse reactions. Post-operative x-ray showed a well-fixed prosthesis (Figure 2) and she was started on intravenous daptomycin 500 mg every day. The morning after the surgery the patient’s aspartate aminotransferase (AST) and alanine aminotransferase (ALT) transiently increased to 1.22 and 1.69 (µkat/L) from a baseline of 0.67 and 1 µkat/L, respectively. The plan was for daily intravenous bacteriophage therapy for 4 days, but she was concerned about further increases in AST and ALT and consequently declined intravenous phage dosing. Liver function returned to her baseline 2 days later where these parameters have remained. Intraoperative cultures taken at time of DAIR grew the same *S. epidermidis* determined by having the exact same sensitivity profile of all her S. epidermidis isolates (Table 1) which had previously grown on all her prior intraoperative and arthrocentesis cultures. In one culture, rapid growth of *Serratia marcensens* occurred even though gram stain did not show Gram-negative rods but rather only Gram-positive cocci. This was considered to be a contaminant, but given her need for aggressive chemotherapy, intravenous ertapenem 1 g daily was used in addition to intravenous daptomycin 500 mg daily for 6 weeks. Her post-operative course was uneventful, and she was discharged home after 5 days in the hospital.

Oral rifampin started 1 week after DAIR but was stopped given symptomatic nausea which resolved after cessation of therapy. After 6 weeks of intravenous antibiotics the patient was transitioned to oral doxycycline 100 mg po bid. After the surgery, the patient went from requiring biweekly blood product transfusions to not requiring blood product transfusions (Figure 1); her moderate leukopenia persisted, with an average white blood cell count of 3 × 10^9^ cells/L (ref: 4.3–10.8 cells/L). This encouraged the patient and her oncologist that her aplastic anemia was recovering, and the decision was made to not have the patient undergo aggressive chemotherapy. Consequently, doxycycline has been continued given its anti-inflammatory properties and to preserve the recovery that has occurred with respect to her underlying pancytopenia. Five months after DAIR and adjuvant bacteriophage therapy, the patient has full range of motion of her knee and no clinical signs of PJI recurrence.

## 3. Methods

### 3.1. Bacterial Isolation

The *S. epidermidis* bacterial isolate was obtained from a sterile arthrocentesis in which this organism was cultured from the synovial fluid. Sensitivities of the *S. epidermidis* can be seen in Table 1. No other pathogens were recovered from this procedure. The clinical isolate was then subcultured onto a tryptic soy agar slant and sent to PhagoMed to identify an appropriate bacteriophage for therapeutic use.

### 3.2. Bacteriophage Screening

**Figure 3:**
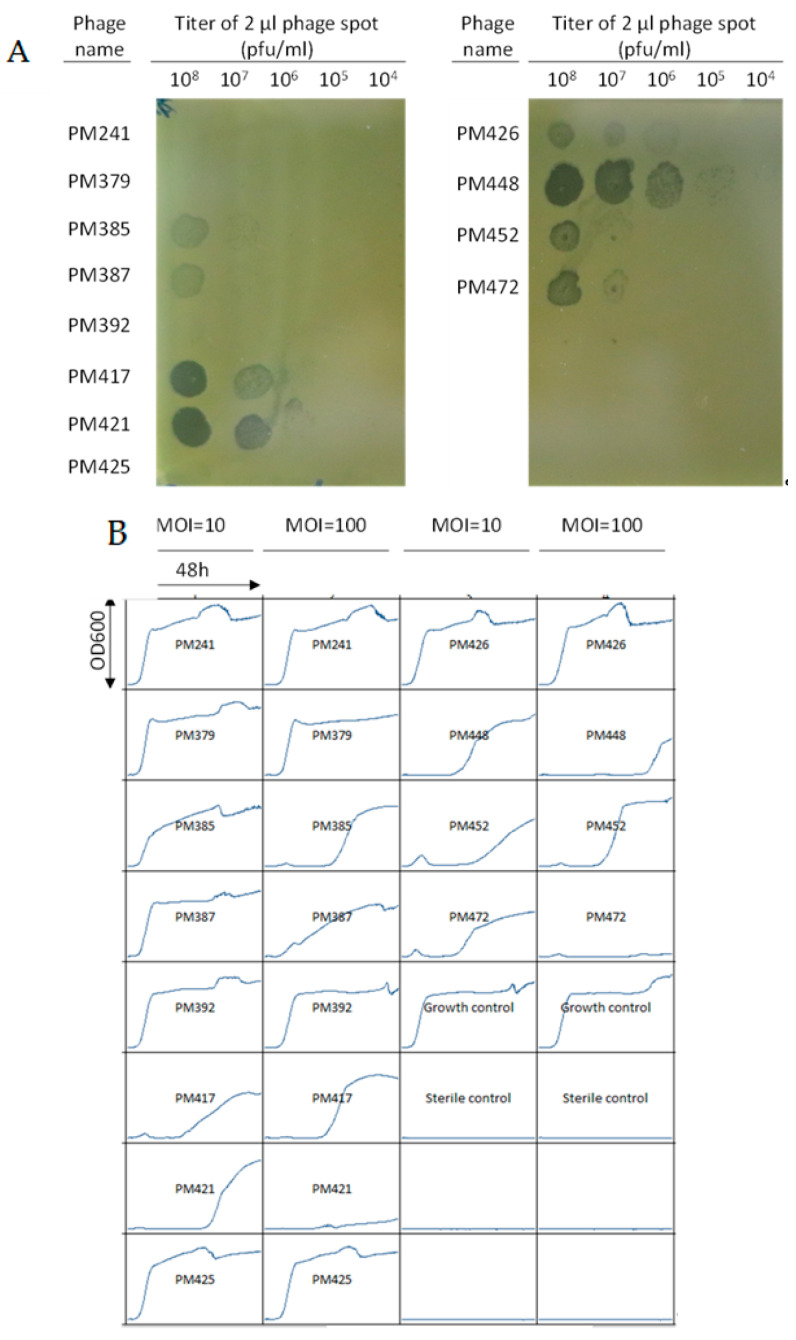
(**A**) Plaquing test by the BHI agar overlay method. A total of 2 μL of the ɛ^2^-phages indicated on the left at 10^8^ pfu/mL were spotted onto a top agar layer containing 10^8^ colony forming units/mL of this patient’s *S. epidermidis* isolate. (**B**) “Growth inhibition” curves for the same ɛ^2^-phages as in (**A**) with the various bacteriophages to this patient isolates *S. epidermidis* clinical isolate. Different bacteriophages are indicated by PM241-PM472. MOI refers to multiplicity of infection. Growth control is the clinical isolate with no bacteriophages. Each box has time on the *x*-axis with end point being 48 h and the optical density (OD) at 600 nm on the *y*-axis.

Only one wild-type phage of the Herelleviridae family formed plaques on the clinical isolate, but could not inhibit growth of the bacteria in suspension at a multiplicity of infection (MOI) of 10 or 100 (data not shown). Several ɛ^2^-phages were also tested, which are bred phages, generated at PhagoMed by superinfecting *S. epidermidis* strains with multiple wild type phages. This induces random homologous recombination, and subsequent selection generated bacteriophages with increased virulence, host range and reduced resistance formation. One such ɛ^2^-phage, PM448, formed plaques at an efficiency of plaquing (EOP) of 800% (Figure 3A) and inhibited bacterial growth at a MOI of 100 for 40 h (Figure 3B). Two other ɛ2-phages, PM421 and PM472, were also able to suppress growth substantially compared to the growth control (Figure 3B). However, each of PM448, PM421 and PM472 allowed the formation of bacteriophage-insensitive mutants (BIM) within 24–48 h, as confirmed by streaking and spot-testing the outgrowth from the wells. The three phages must be similar to each other, as the BIMs from each phage were cross-resistant to the two other phages (data not shown). Therefore, a cocktail would not have been advantageous. The minimum rate of resistance formation of each phage on this patient strain was 1 cell in 2 × 10^5^, the starting number of cells in the MOI = 100 group. While the BIMs from PM421 and PM472 may have grown more slowly than the BIMs of PM448 in this experiment, the rate of BIM formation is likely similar for the three phages, as in each case BIMs start to be detectable by OD600 measurement after ~24 h (Figure 3B). However, the EOP of phage PM448 on the patient strain (800%, Figure 3A) is 9 times and 67 times higher than the EOPs of PM421 (85%) and PM472 (12%), respectively. This indicates that PM448 can infect the patient strain more efficiently and might lead to a higher amount of killed cells than PM421 or PM472, which is why it was selected for the treatment. PM448 was genomically sequenced to confirm absence of lysogeny, pathogenicity genes or antimicrobial resistance genes.

### 3.3. Phage Amplification, Purification and Quality Control Testing

Amplification of Phage PM448 was conducted on the *S. epidermidis* strain isolated from the patient. Briefly, the host strain was grown to the stationary phase using tryptic soy broth of non-animal origin (Sigma Aldrich, St. Louis, MO, USA), then back diluted to 1:1000 and infected with phage PM448 at a MOI of 0.01. Phage were allowed to amplify for 6 h at 37 degrees in a shaker incubator (200 rpm) until the culture cleared. The lysate was then filtered with PVDF filters (Millipore, pore size: 0.22 μm) and immediately concentrated with centrifugal concentrators (amikon, 100 kDa cutoff) to a final titer of 1 × 10^13^ PFU/mL. Titer was measured by traditional double layer agar overlay on the clinical isolate. This concentrate was diluted 1:1000 in PBS with MgSO4 (10 mM) for therapeutic application with titers of 1 × 10^10^ PFU/mL. Results of endotoxin units/mL and sterility testing with USP-71 is displayed in Table 2. Endotoxin was determined using ENDONEXT™ assay (Biomerieux, Marcy l’Etoile, France).

### 3.4. DAIR Procedure and Bacteriophage Administration

The patient underwent a true DAIR procedure in which after induction of anesthesia a midline incision was made directly over her prior well-healed incision. Debridement of infected tissues and synovium was thoroughly conducted from all compartments of the knee. After debridement, cultures were obtained followed by removal of the infected polyethylene liner. Then, with the use of a sterile scrub brush (no detergent) all of the exposed surfaces of metal were extensively scrubbed to break up the biofilm. Pulsatile lavages with normal saline were then used to lavage the knee joint extensively. After this, new gloves, drapes and instruments were applied and then a new polyethylene liner was placed. The knee was then sutured but a superficial drain was placed to help with complications of bleeding given the patients underlying severe pancytopenia.

The bacteriophage was prepared by withdrawing 2 mL from a stock solution of 10 mL of bacteriophage PM448 that had titers of 1 × 10^10^ PFU/mL with an 18-guage needle. The 2 mL were then mixed with 8 mL of normal saline and placed into a 10 mL sterile syringe. This was conducted in a biologic hood under strict sterile conditions. An 18-guage needle was placed into the patient’s intraarticular space. The syringe filled with bacteriophage therapy was then attached to this needle and injected slowly into the intraarticular space. The needle was removed, and the patient was transported to post anesthesia care unit without any complications.

## 4. Discussion

When chronic PJIs fail the gold standard 2-stage revision surgery they are considered recalcitrant and success with further standard of care surgical interventions can be limited [4,5,11]. This is secondary to multiple factors, including but not limited to: comorbidities, underlying immunodeficiencies, virulence of pathogen and residual niduses of chronic deep-seated infection that conventional antibiotics have limited ability to eradicate [4,5,11,12]. In this case, our patient had failed a previous two-stage revision surgery and her severe pancytopenia limited our surgical options to cure her infection. In addition, the high mortality associated with untreated aplastic anemia did not allow for more conservative management with chronic suppression therapy which regardless did not help her after 6 months of oral doxycycline therapy. Rather, eradication of her infection was paramount to then be able to improve her aplastic anemia with chemotherapy to prolong her life. Consequently, the patient consented to receive experimental adjuvant bacteriophage therapy to be used with DAIR to help eradicate the deep-seated biofilm infection as have occurred in other case reports [3,6,7,8].

Using DAIR for the treatment of chronic PJIs has varying success with conservative success rates around 50–55% [13]. These poor success rates are secondary to the limited ability of this surgical technique in combination with conventional antimicrobials to eradicate the deep-seated biofilm in chronic PJIs. Therefore, in the treatment of chronic PJIs, DAIR is not widely used except in patients that have well fixed prosthetics and have either symptoms of PJIs for less than 3 weeks or comorbidities that at the judgement of the orthopedic surgeon severely limit the ability to conduct two-stage revision surgery [14]. When using adjuvant bacteriophage therapy in the treatment of PJIs, DAIR does have some advantageous aspects that may allow cure of these chronic infections without need for removal of the prosthesis. These benefits include (1) surgical removal of the overlying planktonic infection, (2) ensuring the prosthesis is fixed and can be salvaged, (3) obtaining intraoperative cultures to ensure all pathogens are identified, (4) exchange of modular components, (5) manual debridement of the biofilm and (6) direct application of bacteriophages to the infected prosthesis that harbors the bacterial biofilm that was recently manually debrided. This initiates a bacteriophage infection in the biofilm and, given bacteriophage’s ability to self-replicate and increase its concentration, allows for theoretical stepwise degradation of biofilm [15]. It must be noted that unlike conventional antibiotics, bacteriophages behave differently, and many other parameters (duration of growth inhibition, theoretical multiplicity of infections, rates of resistance development) should be considered to contribute to effective treatment protocols [16].

In this case, the ɛ^2^-bacteriophage used (PM448) formed plaques at a EOP of 800% (i.e., eight times more efficiently than on the *S. aureus* strain on which it was originally propagated), and inhibited in vitro growth of the clinical *S. epidermidis* isolate for 40 h (Figure 2) at a MOI of 100. In contrast, the wild type ancestors of PM448 had EOP values only up to 1% and could not control bacterial growth in suspension. This indicates that the breeding process markedly increased the virulence of PM448 on this *S. epidermidis* patient strain. For the same bacteriophage, PM448, when the MOI was 10 only 24 h growth inhibition occurred (Figure 2). This had implications on our use of this bacteriophage therapy given the unknown distribution of IV phage therapy to the synovium and the dose-dependency seen with in vitro testing displayed in Figure 2 [17]. We therefore elected to use intraarticular administration to achieve maximum local doses in line with previous reports of successful use of bacteriophage therapy in PJI [7,16]. As well, the use of DAIR removed most of the planktonic bacteria and removed a portion of the chronic biofilm by manual debridement thereby reducing the total number of pathogenic bacteria [18]. This caused a favorable theoretical MOI for bacteriophages to infect a chronic biofilm and eradicate it. It has been theorized that to eradicate a biofilm with bacteriophages, high phage doses ≥10^10^ pfu are needed to be effective in this aim [7,19,20]. In correlation, manual debridement of the biofilm and exchange of prosthetic modular parts have benefits in treating PJIs by directly exposing biofilm bacteria to bacteriophages, thus allowing for enhanced attachment resulting in more robust infection of biofilm bacteria [18,21]. Therefore, using bacteriophages with DAIR could improve cure rates sparing patients the need for much more invasive debilitating surgeries that require removal of prostheses which some patients like the one described here is not feasible.

In this case no severe adverse events were seen with a large dose (2 × 10^10^ PFU) of intraarticular bacteriophage therapy other than a transient increase in liver enzymes (2-fold increase compared to baseline liver enzymes). This caused the patient to decline subsequent intravenous phage therapy after her intraarticular dose. It should be noted that our patient did have underlying chronic liver inflammation from her biweekly recurrent blood transfusions for her aplastic anemia and therefore numerous factors could have caused this increase other than bacteriophage therapy. While the temporal relationship is suggestive that bacteriophages may have had some contribution, we have limited support for this claim. As well, it is unknown if continued phage dosing would have caused further transaminitis similar to our experience in a previous case [3]. This case reinforces that prudent monitoring of liver function is needed in patients with underlying liver pathology given the extensive hepatic clearance of bacteriophage therapy and potential hepatocyte inflammation. In correlation, there is a paucity of safety data with the use of high intravenous or intraarticular bacteriophage titers beyond a single phase 1 clinical trial evaluating dose escalation of intravenous bacteriophage therapy [22]. Therefore, further safety studies are needed with these higher titers of bacteriophage therapies.

While we anticipated an improvement in her PJI, it was surprising that our patient had such a dramatic improvement in her aplastic anemia after DAIR and adjuvant bacteriophage therapy (Figure 1). She had previously had no improvement of her pancytopenia with aggressive antibiotic or surgical interventions for her *S. epidermidis* PJI or with oral decitabine and eltrombopag therapies. This recovery likely occurred as a result of adequate treatment of her PJI, which led to the removal of the chronic systemic inflammation, thereby removing a chronic source of prolonged bone marrow suppression. In support of this theory is the known pathophysiology of aplastic anemia in which chronic inflammatory cytokines are correlated with continued stresses leading to bone marrow suppression [23]. Additionally, there are some data to suggest Staphylococcal peptidoglycan may suppress hematopoiesis; a case of acute arrest of hematopoiesis from a disseminated *S. epidermidis* infection has been reported [24,25]. Unique to our case was the lack of observable bacteria on her numerous bone marrow biopsies and her lack of improvement with prior aggressive antibiotics that had activity to her clinical isolate. While her recovery further supports that clinical cure of her PJI likely occurred, the drastic improvement has caused us to continue chronic oral doxycycline to maintain her quality of life and continue to forgo the need for aggressive chemotherapy. This does come with a trade off as with the prolonged use of oral doxycycline this limits our ability to definitely document success of adjuvant bacteriophage therapy in curing her PJI in this case. However, her clinical improvement, previous lack of improvement with long term oral doxycycline therapy and the changes that occurred with respect to her pancytopenia are highly suggestive of cure. This is a limitation similar to other cases that did not have any severe underlying comorbidities but were continued on chronic suppression therapy and were deemed to have successful treatments [6,26].

## 5. Conclusions

In conclusion, this case reinforces that the use of intraarticular bacteriophage therapy as an adjuvant to DAIR and standard of care medical management in recalcitrant PJIs holds promise to improve morbidity and reduce mortality by treating these infections while retaining the prosthesis. However, further translational research is needed to clarify many aspects of this therapeutic, so this technique can be generalizable to wide groups of patients and not be relegated to personalized individual cases as seen here. Therefore, well devised safety and proof of concept trials are warranted to elucidate safety and standardize treatment protocols and parameters before efficacy trials are to be conducted.

## Figures and Tables

**Table 1:** *S. epidermidis* Susceptibility Results.

Antibiotic	MIC µg/mL	Interpretation
Cefazolin	NA	Resistant
Ciprofloxacin	˃2	Resistant
Daptomycin	≤0.25	Sensitive
Erythromycin	˃4	Resistant
Gentamicin	≤1	Sensitive
Linezolid	1	Sensitive
Oxacillin	˃2	Resistant
Rifampin	≤1	Sensitive
Tetracycline	≤2	Sensitive
TMS	˃2/38	Resistant
Vancomycin	1	Sensitive

NA—Not available, TMS—Trimethoprim/Sulfamethoxazole.

**Table 2:** Final therapeutic preparation details provided to this patient for the treatment of *S. epidermidis* PJI infection.

Phage ID	Titer (PFU/mL)	Endotoxin (EU/dose)	USP <71> Sterility Testing
PM448	1 × 10^10^	<1	PASSED

## Data Availability

The data presented in this study are available on request from the corresponding author.
